# Genetic Adjuvantation of Recombinant MVA with CD40L Potentiates CD8 T Cell Mediated Immunity

**DOI:** 10.3389/fimmu.2013.00251

**Published:** 2013-08-27

**Authors:** Henning Lauterbach, Juliane Pätzold, Ronny Kassub, Barbara Bathke, Kay Brinkmann, Paul Chaplin, Mark Suter, Hubertus Hochrein

**Affiliations:** ^1^Department of Research Immunology, Bavarian Nordic GmbH, Martinsried, Germany; ^2^Department of Vaccine Development, Bavarian Nordic GmbH, Martinsried, Germany; ^3^Bavarian Nordic GmbH, Martinsried, Germany; ^4^University of Zurich, Zurich, Switzerland

**Keywords:** vaccination, viral vaccines, CD8 T cells, T cell memory, co-stimulation, modified vaccinia Ankara, CD40L, ectromelia virus

## Abstract

Modified vaccinia Ankara (MVA) is a safe and promising viral vaccine vector that is currently investigated in several clinical and pre-clinical trials. In contrast to inactivated or sub-unit vaccines, MVA is able to induce strong humoral as well as cellular immune responses. In order to further improve its CD8 T cell inducing capacity, we genetically adjuvanted MVA with the coding sequence of murine CD40L, a member of the tumor necrosis factor superfamily. Immunization of mice with this new vector led to strongly enhanced primary and memory CD8 T cell responses. Concordant with the enhanced CD8 T cell response, we could detect stronger activation of dendritic cells and higher systemic levels of innate cytokines (including IL-12p70) early after immunization. Interestingly, acquisition of memory characteristics (i.e., IL-7R expression) was accelerated after immunization with MVA-CD40L in comparison to non-adjuvanted MVA. Furthermore, the generated cytotoxic T-lymphocytes (CTLs) also showed improved functionality as demonstrated by intracellular cytokine staining and *in vivo* killing activity. Importantly, the superior CTL response after a single MVA-CD40L immunization was able to protect B cell deficient mice against a fatal infection with ectromelia virus. Taken together, we show that genetic adjuvantation of MVA can change strength, quality, and functionality of innate and adaptive immune responses. These data should facilitate a rational vaccine design with a focus on rapid induction of large numbers of CD8 T cells able to protect against specific diseases.

## Introduction

Decades of successful vaccine development led to a drastic reduction in child mortality and debilitating illness resulting from infectious disease. Despite this impressive success, probably best exemplified by the eradication of smallpox, an estimated 5.6 million deaths annually can be accounted solely to the three most devastating diseases: malaria, tuberculosis, and HIV/AIDS (1). Other infectious diseases, such as Ebola and Marburg hemorrhagic fever, are quite rare but accompanied by high fatality rates. Moreover, the corresponding viruses might be used as bioterrorism agents. The common element linking these very diverse diseases is the requirement for strong T cell responses as part of the adaptive immune response to protect the host from infection. Because it is generally accepted that an ideal vaccine should induce both, T cells and antibodies, modern vaccinology is faced with the challenge to create a safe vaccine able to activate efficiently both arms of the adaptive immune system. Among the many possible vaccine modalities, live vector viruses seem best to fulfill this requirement (2). While some live virus vaccines, such as vaccinia virus (VV) and yellow fever virus are effective but have unfavorable safety profiles, others, such as adenovirus (AdV), face problems due to pre-existing immunity [reviewed in (3)]. A safe live vector vaccine largely unaffected by pre-existing immunity (4–6) is modified vaccinia virus Ankara (MVA), originally created by Anton Mayr and further developed into a third-generation smallpox vaccine (MVA-BN^®^, IMVAMUNE^®^) [reviewed in (7)].

The excellent safety profile of MVA, which stems from its replication deficiency in human cells, has been proven in many clinical trials, including vaccination of immune-compromised individuals, and during the smallpox eradication campaign in the 1970s, when 120,000 people were vaccinated with MVA (8). Since then, many different recombinant MVA vaccines have been generated and tested for the ability to immunize animals and humans against infectious (e.g., HIV, malaria) and non-infectious (e.g., prostate cancer) diseases. Its proven safety and good immunogenicity thus makes MVA a prime candidate for a T and B cell-inducing vaccine vector.

Classically, vaccine development has focused on the induction of neutralizing antibodies. In recent years, however, T cells, especially CD8 T cells, have moved into the focus of vaccinologists. This paradigm shift stems from an increasing amount of data, showing the need for high amounts of multi-functional CD8 T cells to clear or protect against pathogens such as HIV/SIV (9, 10), Ebola virus (11), human papillomavirus (HPV) (12), hepatitis C virus (HCV) (13), and *Plasmodium falciparum*, the causative agent of malaria (14, 15). Furthermore, adoptive transfer experiments have demonstrated the therapeutic potential of CD8 T cells in treating cancer (14, 16). This increased awareness of the importance of CD8 T cells has spurred more basic science aimed at better understanding T cell biology. Even though not all factors and mechanisms governing cytotoxic T-lymphocyte (CTL) expansion and memory differentiation are understood in all details, it is believed that expansion, development of effector functions, and ultimately, differentiation into memory cells are mainly determined during the first few days after antigen encounter (17). In a simplistic view, antigen recognition via the T cell receptor (TCR) (signal 1) activates the T cell and co-stimulation via CD28 and CD80/CD86 (signal 2), and the secretion of cytokines such as interleukin-12 (IL-12) and interferon-α (IFN-α) (signal 3) ensure the efficient generation of effector and memory CTLs. However, after pathogen encounter a plethora of additional stimulatory and inhibitory molecules are induced, creating a specific inflammatory environment, which further influences quality and quantity of CTL responses (18).

Among those molecules, members of the tumor necrosis factor receptor/tumor necrosis factor (TNFR/TNF) superfamily are well known for their T cell shaping properties. This family includes, among others, CD27/CD70, CD30/CD30L, CD40/CD40L, OX40/OX40L, 4-1BB/4-1BBL, GITR/GITRL, and Fas/FasL. The role of CD70, OX40L, and 4-1BBL for primary and secondary T cell responses was investigated in a broad range of infectious disease models (19–21). Interestingly, the up-regulation of co-stimulatory molecules (including CD80, CD86, CD70, 4-1BBL, and OX40L) on dendritic cells (DCs) can be induced by combined Toll-like receptor (TLR)/CD40-stimulation (22). Furthermore, TLR/CD40 ligation also induces the expression of pro-inflammatory cytokines, including IL-12p70, by DCs *in vivo* (23). Thus, CD40 can be regarded as a master-switch for DC activation. While CD40 is constitutively expressed on many cell types, including B cells, macrophages, and DCs, its ligand CD40L is predominantly expressed on activated CD4 T cells (24, 25). The cognate interaction between DCs and CD4 T cells early after infection or immunization ‘licenses’ DCs to prime CD8 T cell responses (26, 27). DC licensing results in the up-regulation of co-stimulatory molecules, increased survival and better cross-presenting capabilities of DCs. This process is mainly mediated via CD40/CD40L interaction (28, 29), but CD40/CD40L-independent mechanisms also exist (30, 31). Interestingly, the direct interaction between CD40L expressed on DCs and CD40 expressed on CD8 T cells has also been suggested, providing a possible explanation for the generation of helper-independent CTL responses (32).

Several studies indicate that agonistic anti-CD40 antibody (Ab) may be useful as a vaccine adjuvant. In addition, recombinant AdV (33) and VV (34) encoding CD40L have been created that showed superior immunogenicity *in vitro* and *in vivo* compared to non-adjuvanted viruses. Based on these data, the central role of CD40/CD40L co-stimulation for CD8 T cell responses and the good CTL-inducing capacities of MVA together with its favorable safety profile, we constructed a recombinant MVA expressing CD40L and the model antigen ovalbumin (OVA). *In vitro* and *in vivo* analyses revealed significantly enhanced DC activation and cytokine production (including high levels of IL-12p70) after treating cells or mice with MVA-OVA-CD40L. This effect was entirely dependent on *de novo* CD40L gene expression, partly contradicting previous results (34). While Ab responses were not increased, immunization with MVA-OVA-CD40L led to strongly enhanced primary and memory CD8 T cell responses. Of note, one immunization with MVA-OVA-CD40L induced the same number of antigen-specific CTL as two immunizations with MVA-OVA. Importantly, not only the quantity but also the quality of the CTL response was improved, as revealed by intracellular cytokine staining and *in vivo* killing activity. Finally, the superior T cell response directly translated into better protection against a fatal virus infection (mousepox) in B cell deficient mice. These results highlight the potential of a CD40L-adjuvanted MVA to induce rapidly strong antigen-specific multi-functional CD8 T cell responses. Thus, recombinant MVA-CD40L is a prime candidate for the development of prophylactic and therapeutic vaccines against diseases such as cancer, HIV/AIDS, Ebola and Marburg hemorrhagic fever, malaria and hepatitis C, and also for emergency vaccinations in cases of bioterrorism attacks.

## Results

### MVA-induced CD8 T cell responses are amplified by an agonistic anti-CD40 antibody

**Figure 1:**
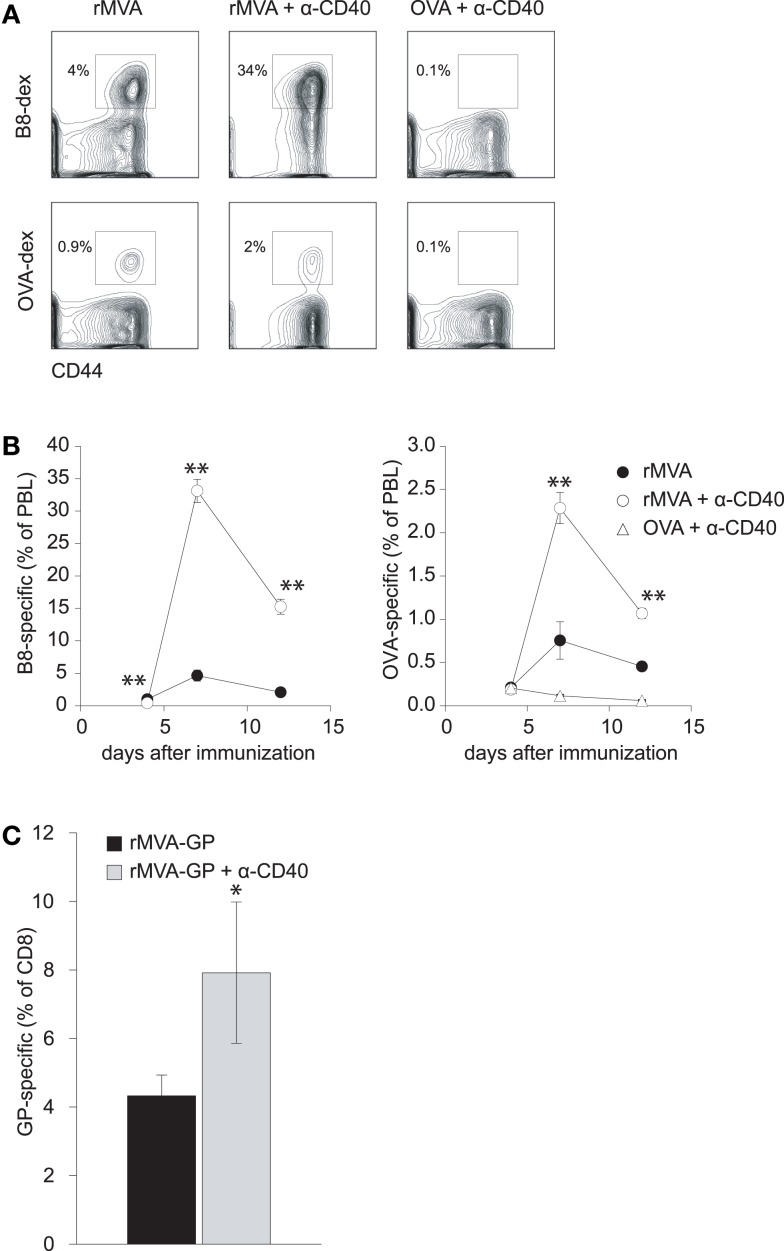
**CD40 co-stimulation increases CTL responses to rMVA encoded antigens but not to a protein antigen**. C57BL/6 mice were immunized with rMVA, rMVA + anti-CD40 mAb, or OVA protein + anti-CD40 mAb. B8- and OVA-specific CD8 T cells were visualized by MHC class I dextramer staining. **(A)** Density plots illustrate the frequency of B8- and OVA-specific CTL in the blood on day 7 (gated on CD8^+^ PBL). Numbers indicate the percentage of CD8^+^ dextramer^+^ T cells among PBL. A kinetic analysis of B8- and OVA-specific CTLs is shown in **(B)**. CTL responses to B8 and OVA were drastically enhanced by co-injection of rMVA and anti-CD40 mAb. Note, that no detectable CTL response was generated after immunization with OVA protein and anti-CD40 antibody. **(C)** Similar to the above described experiment, CBA/J (H-2k) mice were immunized with rMVA-GP encoding the glycoprotein of Zaire Ebola virus ± anti-CD40 mAb. The GP-specific CD8 T cell response was measured 7 days later by intracellular cytokine staining. Shown is the mean percentage ± SEM of IFN-γ^+^ GP-specific CD8 T cells in the spleen. Data are representative of four mice per group.

The combination of a TLR-ligand and a CD40 agonist has been shown to synergistically enhance antigen-specific CD4 and CD8 T cell responses after protein immunization (35, 36). Because of the TLR-stimulating properties of MVA (37, 38), we hypothesized that co-administration of MVA and a CD40 agonist might lead to enhanced CD8 T cell responses. Therefore, we first set out to evaluate whether MVA-induced CD8 T cell responses can be amplified by an agonistic Ab to murine CD40. Mice were immunized with MVA-OVA (hereafter referred to as rMVA), rMVA mixed with anti-CD40 Ab, or OVA protein combined with anti-CD40 Ab. MHC class I (H-2Kb) dextramers loaded with either B8_20–27_- or OVA_257–264_-peptide were used to detect MVA- and OVA-specific CD8 T cell responses, respectively (Figure 1A). Flow cytometric analysis of peripheral blood lymphocytes (PBL) revealed that rMVA immunization induced B8- and OVA-specific CD8 T cell responses. These responses were enhanced ∼7- and ∼3-fold, respectively, by anti-CD40 Ab (Figure 1B). OVA/anti-CD40 immunization, in contrast, did not lead to a detectable antigen-specific CD8 T cell response. In order to verify that our findings with the model antigen OVA are transferable to pathogen-derived antigens, we repeated the above experiment using recombinant MVA encoding the glycoprotein (GP) from Zaire Ebola virus (rMVA-GP). Ebola GP-specific CD8 T cells were detected by intracellular cytokine staining after re-stimulation with the H-2Kk restricted peptide GP_577–584_ (39). Again, we could observe a significantly (*p* < 0.05) enhanced GP-specific CD8 T cell response in the spleen of mice immunized with rMVA-GP + anti-CD40 (Figure 1C).

### Construction and *in vitro* characterization of rMVA-CD40L

**Figure 2:**
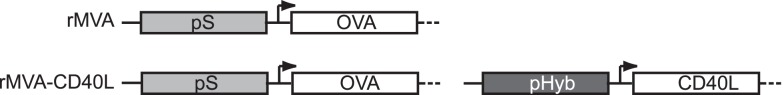
**Schematic representation of rMVA and rMVA-CD40L**. Recombinant MVA encoding OVA alone or OVA together with murine CD40L were generated as described in Materials and Methods. OVA expression is controlled by the early/late synthetic promoter (pS) and CD40L expression by the recently described early/late hybrid promoter (pHyb) (40).

In order to take advantage of the large transgene acceptance of MVA, we decided to create a recombinant MVA encoding both, the neo-antigen and the co-stimulatory molecule. To this end, a CD40L expression cassette including the recently described early/late hybrid promoter (pHyb) (40) and the cDNA sequence of murine CD40L was inserted into a recombinant MVA expressing OVA under the control of the early/late synthetic promoter (pS) (41) (Figure 2). This promoter choice guaranteed a simultaneous expression of antigen and co-stimulus. The model antigen OVA instead of Ebola GP was used because more analytical tools are available for OVA and the above described experiments suggested that OVA based findings were likely to be applicable to other neo-antigens as well.

**Figure 3:**
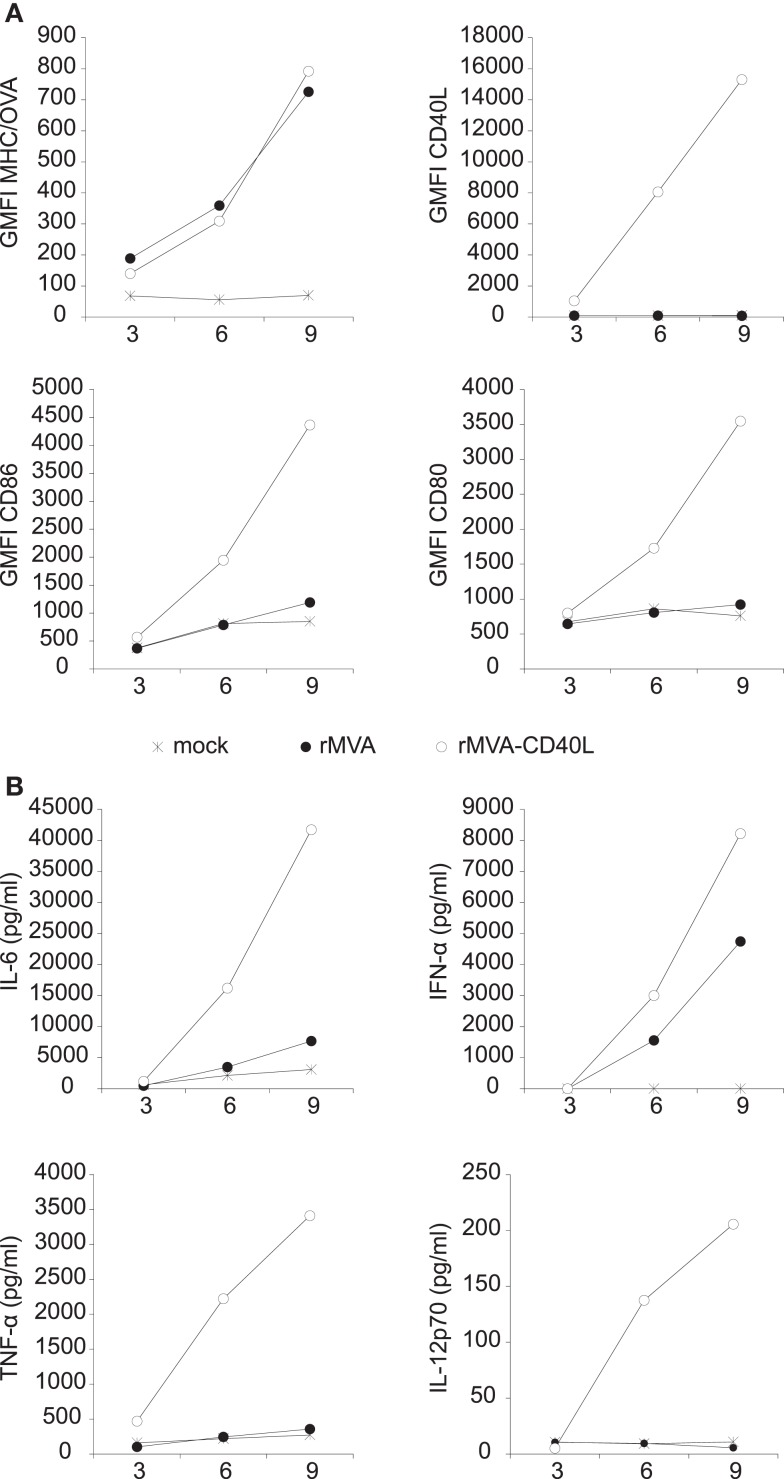
**Transduction of FLDCs *in vitro* with rMVA-CD40L leads to superior DC activation and cytokine secretion**. *In vitro* generated FLDCs were transduced with rMVA or rMVA-CD40L (MOI2). **(A)** Three, six, and nine hours after transduction DCs were analyzed for OVA (H-2Kb/OVA_257–264_ complexes) and CD40L expression and the activation markers CD86 and CD80. Note that OVA expression is not different between rMVA and rMVA-CD40L. **(B)** At the same time points as in **(A)** supernatants were analyzed for IL-6, IFN-α, TNF-α, and IL-12p70 by a cytometric bead assay. Note that IL-12p70 and TNF-α were only detectable after transduction with rMVA-CD40L but not rMVA. Data are representative of at least two independent experiments. Shown is the mean of duplicate samples.

The expression of OVA and CD40L was verified by transducing FLT3-L bone marrow culture derived DCs (FLDCs) with rMVA or rMVA-CD40L. OVA expression was measured by using the 25-D1.16 Ab that specifically detects H-2Kb/OVA_257–264_ complexes (42). As shown in Figure 3A (upper row), OVA expression was readily detected after 3 h and continued to increase during the observation period of 9 h. While there was no difference in OVA expression in rMVA or rMVA-CD40L transduced cells, CD40L was exclusively expressed in cells transduced with the latter. Importantly, H-2Kb/OVA_257–264_ complexes and CD40L appeared synchronously on the cell surface of transduced FLDCs, ensuring the isochronic availability of antigen and co-stimulus. The same cells were also analyzed for expression of the two activation markers CD86 and CD80. rMVA transduced FLDCs showed only weak signs of activation after 9 h compared to untreated cells (Figure 3A; bottom row). In contrast, the geometric mean fluorescence intensity (GMFI) of CD86 and CD80 was 3- to 4-fold higher after 9 h in rMVA-CD40L transduced cells compared to rMVA transduced cells. In order to further compare the *in vitro* immunogenicity of the two constructs, the amount of secreted cytokines in the supernatant of transduced FLDCs was measured. Both, rMVA and rMVA-CD40L transduced cells secreted IL-6 and IFN-α above background levels, with far higher levels in rMVA-CD40L transduced cells (Figure 3B). TNF-α and IL-12p70, however, were strongly expressed exclusively by rMVA-CD40L transduced FLDCs. Thus, the addition of CD40L into rMVA did not change OVA-antigen expression but improved its immunogenicity *in vitro*.

### Heightened DC activation and innate cytokine production *in vivo* after immunization with rMVA-CD40L

**Figure 4:**
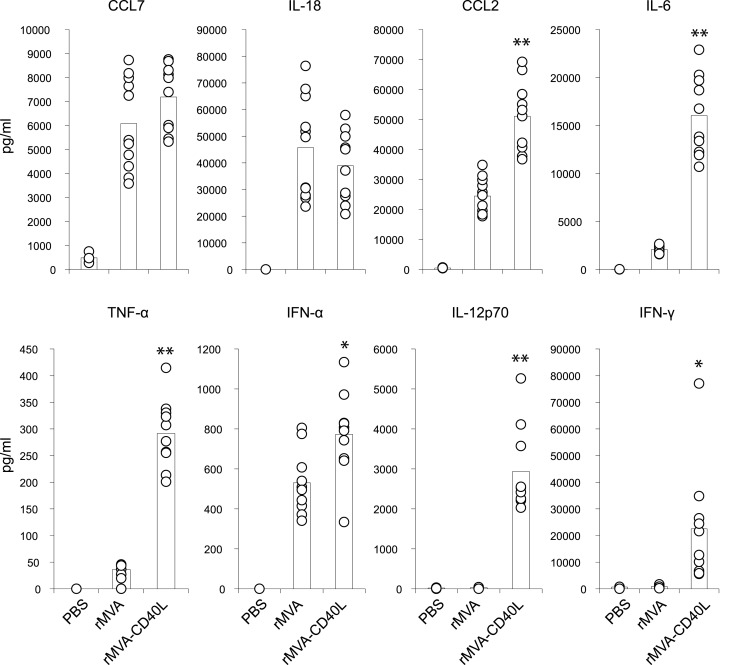
**Enhanced systemic cytokine levels after injection of rMVA-CD40L**. C57BL/6 mice were treated with PBS, rMVA, or rMVA-CD40L. Six hours after injection, serum cytokine levels were determined by a cytometric bead assay. Note that IL-12p70 was only detectable after rMVA-CD40L injection. IFN-γ levels were low to undetectable after rMVA immunization. Results are compiled from three independent experiments. Circles denote individual mice and bars the mean of all mice per group.

**Figure 5:**
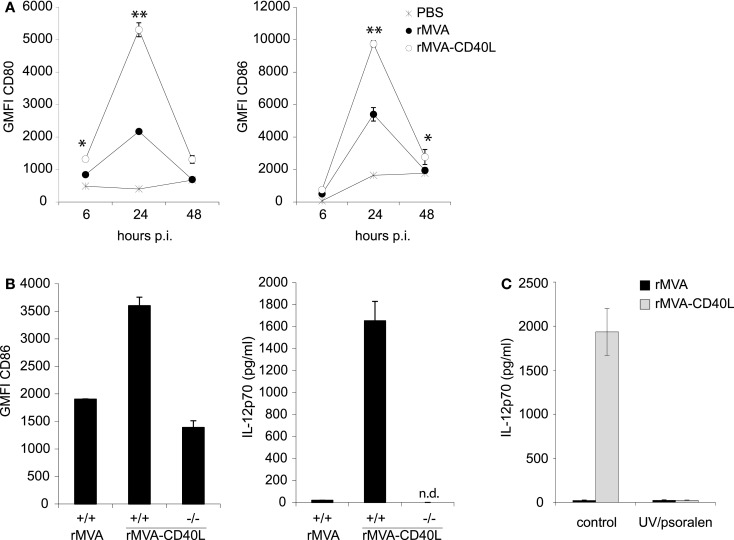
**Enhanced DC activation *in vivo* after rMVA-CD40L immunization**. C57BL/6 mice were treated with PBS, rMVA, or rMVA-CD40L. **(A)** Six, twenty four, and forty eight hours after injection splenic DCs were analyzed for expression of the activation markers CD80 and CD86. Similar to *in vitro* transduced DCs (see Figure 3), splenic DCs were more strongly activated by rMVA-CD40L. The peak of activation was at 24 h post immunization. **(B)** In order to test whether the enhanced immunogenicity of rMVA-CD40L is dependent on the interaction of CD40 and virally encoded CD40L, we immunized CD40^+/+^ C57BL/6 mice with rMVA or rMVA-CD40L and CD40^−/−^ mice with rMVA-CD40L. Serum concentration of IL-12p70 was determined 6 h after injection and CD86 expression was analyzed on splenic DCs after 24 h. **(C)** C57BL/6 mice were immunized with active or UV/psoralen-inactivated rMVA and rMVA-CD40L. Serum concentration of IL-12p70 was determined 6 h after injection and CD86 expression was analyzed on splenic DCs after 24 h. Shown is the mean ± SEM of three to six mice per group.

To gain insight into the immunostimulatory properties of the two vaccine vectors *in vivo*, mice were intravenously (i.v.) inoculated with rMVA and rMVA-CD40L. Six hours after injection, mice were bled for serum cytokine analyses. While there were no differences in expression of CCL7 and IL-18, rMVA-CD40L induced significantly higher levels of CCL2, IL-6, TNF-α, IFN-α, IL-12p70, and IFN-γ, with the latter two not detectable in serum of rMVA immunized mice (Figure 4). Of note, IL-12p70 levels after rMVA-CD40L immunization were ∼10-fold higher than after injection of the prototypic IL-12 inducer CpG1668 (2936 ± 1046 vs. 292 ± 31 pg/ml). Similar to our *in vitro* analyses, DC activation *in vivo* was also monitored. CD80 and CD86 expression by conventional DCs (cDCs) was increased after rMVA immunization with the peak at 24 h (Figure 5A). Both, CD80 and CD86, were expressed at a significantly higher level after injection of rMVA-CD40L. In order to confirm that the enhanced immunogenicity of rMVA-CD40L is due to the insertion of CD40L, wild-type (wt) C57BL/6 mice were immunized either with rMVA or rMVA-CD40L, CD40^−/−^ mice were immunized with rMVA-CD40L, and CD86 expression by cDCs was measured again after 24 h. While wt DCs expressed higher levels of CD86 following rMVA-CD40L immunization compared to rMVA immunization, there was no enhanced expression by DCs isolated from CD40^−/−^ mice (Figure 5B). Similarly, the cytokine expression pattern after 6 h was comparable between rMVA immunized wt mice and rMVA-CD40L immunized knock-out (ko) mice (Figure 5B). Because Bereta et al. suggested that CD40L might be present in recombinant VV virions (34), it was possible that this might also be true for our vector. To test this possibility, we inactivated rMVA-CD40L by psoralen/UV treatment and measured serum cytokine levels 6 h after immunization. In contrast to active rMVA-CD40L, no IL-12p70 was detected in the serum of mice treated with inactivated virus (Figure 5C). Taken together, these data confirmed the enhanced capacity of rMVA-CD40L to activate innate immunity and proved that the observed effect was strictly dependent on interaction between CD40 and virally encoded and newly expressed CD40L.

### rMVA-CD40L immunization induces high numbers of antigen-specific CD8 T cells and faster memory differentiation

**Figure 6:**
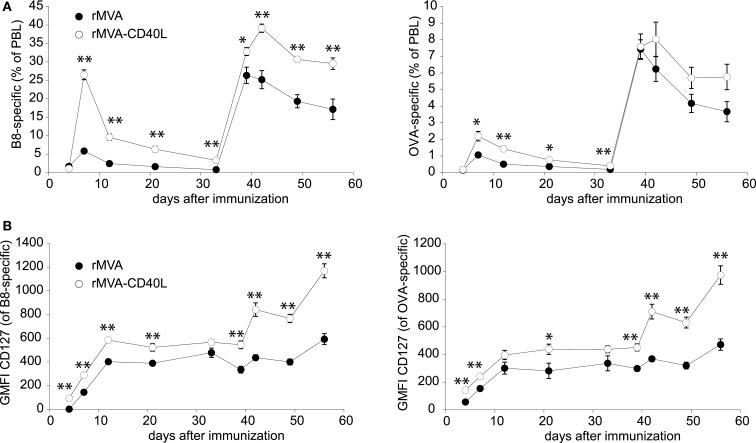
**Increased CD8 T cell response after rMVA-CD40L immunization**. C57BL/6 mice were immunized with rMVA and rMVA-CD40L on day 0 and 35. The primary and secondary B8- and OVA-specific CD8 T cell response was quantified by dextramer staining of PBL at the indicated time points as shown in Figure 1A. **(A)** The kinetic analysis shows the mean percentage ± SEM of CD8^+^ dextramer^+^ T cells among PBL. **(B)** B8- and OVA-specific CD8 T cells were also analyzed flow cytometrically for surface expression of CD127. Data are represented as the mean ± SEM GMFI. Data are representative of five mice per group and two independent experiments.

The strong up-regulation of co-stimulatory molecules on DCs and the induction of high levels of T cell instructing cytokines (e.g., IL-12p70) following rMVA-CD40L immunization suggested that this vector might induce superior CTL responses, too. Therefore, after having assessed innate immunity *in vitro* and *in vivo*, the analyses were extended to examine T cell responses after rMVA-CD40L immunization. To this end, C57BL/6 mice were injected with rMVA or rMVA-CD40L and the frequency of B8- and OVA-specific CD8 T cells in blood over time was measured. In mice of both groups, specific CD8 T cells became detectable on day 4 and peaked on day 7 after the first immunization (Figure 6A). In line with the pilot experiment (see Figure 1), the frequency at the peak of the primary response of B8- and OVA-specific CTLs was ∼5- and ∼2-fold higher, respectively, after rMVA-CD40L immunization. As expected, both T cell populations increased substantially after the boost on day 35 and once again antigen-specific CD8 T cell frequencies were higher in rMVA-CD40L immunized mice. Notably, a single immunization with rMVA-CD40L resulted in the same frequency of B8-specific T cells (∼26% of PBL) as two immunizations with rMVA. In order to gain insight into the effect of additional CD40L stimulation on memory differentiation, we further analyzed the expression of the IL-7 receptor α chain (IL-7Rα, CD127) on antigen-specific CD8 T cells. Surface expression of CD127 on activated CD8 T cells is used as a marker to distinguish between effector and memory T cells (43, 44) and CD127high memory CD8 T cells have been correlated with better protection against bacterial challenge (44). Interestingly, at all time points analyzed B8- and OVA-specific CD8 T cells in rMVA-CD40L immunized mice had higher GMFIs of CD127 (Figure 6B). The differences were not statistically significant at all days, however. The higher GMFI was a result of both, more CD127high cells and a higher CD127 expression on a per cell basis. The difference between the two groups became most prominent after the second immunization. Thus, rMVA-CD40L immunization not only leads to enhanced CTL responses but also to faster memory differentiation.

### Higher expression of IL-2Rα and IL-7Rα on T cells and faster proliferation after rMVA-CD40L immunization

**Figure 7:**
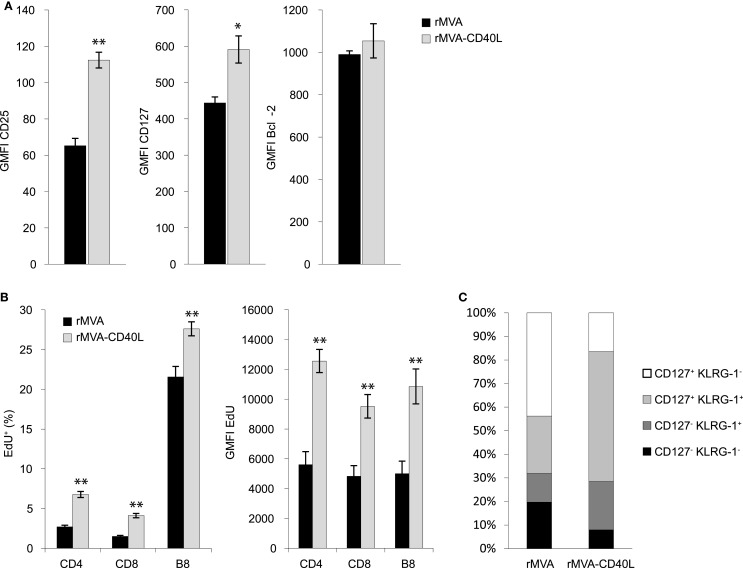
**rMVA-CD40L immunization influences the phenotype and the proliferation rate of CD8 T cells**. C57BL/6 mice were immunized with rMVA and rMVA-CD40L. Five days later expression of CD25, CD127, and Bcl-2 in splenic B8-specific CD8 T cells was analyzed flow cytometrically. **(A)** Bars represent the mean ± SEM GMFI (gated on B8-dextramer^+^ CD8 T cells). In order to measure cell proliferation, mice were treated with the thymidine analog EdU on day 5 after immunization. EdU incorporation into splenic T cells was determined flow cytometrically after 1 h. **(B)** The percentage and GMFI of EdU^+^ CD4, CD8, and B8-specific T cells are shown as the mean ± SEM. **(C)** depicts the relative frequencies of CD127^+^ KLRG-1^−^, CD127^+^ KLRG-1^+^, CD127^−^ KLRG-1^+^, and CD127^−^ KLRG-1^−^ cells among all splenic B8-specific CD8 T cells on day 7 after immunization. Data are representative of five mice per group and at least two independent experiments.

The strongly enhanced CD8 T cell response after rMVA-CD40L immunization prompted us to investigate in more detail the CD8 T cells generated early after priming. Enhanced CD8 T cell numbers can be the result of faster proliferation, less apoptosis, or a combination of both. Proliferation and survival in turn are regulated, at least in part, by cytokines of the IL-2 family (IL-2, IL-4, IL-7, IL-9, IL-15, and IL-21) (45). Especially, enhanced signaling via the IL-2 receptor leads to stronger expansion of antigen-stimulated CD8 T cells (46–48). Therefore, we initially measured expression of CD25 (IL-2Rα), CD127 (IL-7Rα), and the anti-apoptotic molecule Bcl-2 on B8-specific CD8 T cells 5 days after immunization with rMVA or rMVA-CD40L. B8-specific CD8 T cells in rMVA-CD40L immunized animals displayed higher expression of CD25 and CD127 but not of Bcl-2 compared to rMVA immunized mice (Figure 7A). In order to measure cell proliferation, the thymidine analog EdU was administered intravenously on day 5 after immunization and the spleens were excised 1 hour later. As shown in Figure 7B, EdU was incorporated into the DNA of CD4 and CD8 T cells. No EdU uptake was observed in naïve mice (data not shown). The percentage of EdU^+^ CD4 and CD8 T cells was ∼2.5 times higher in rMVA-CD40L than in rMVA immunized mice. Also, significantly more B8-specific CD8 T cells had taken up EdU within 1 h (27.6 ± 0.9% vs. 21.6 ± 1.3%; *p* < 0.005). Furthermore, we determined the amount of incorporated EdU on a per cell basis by measuring the GMFI of EdU^+^ cells. All investigated T cell populations (CD4, CD8, and B8-specific CD8) in rMVA-CD40L immunized mice showed drastically higher GMFIs of EdU, indicating a faster cell cycle. Taken together, both, higher expression of CD25 and CD127 and more and faster proliferating T cells, were observed after immunization with rMVA-CD40L. Because IL-12 is known to influence the memory potential of CD8 T cells (34, 49), we sought to analyze the memory related phenotype of B8 CD8 T cells. As shown in Figure 7C, 12.2 ± 1.6% of B8-specific CD8 T cells had the phenotype of short lived effector cells (SLECs, which are CD127^−^ KLRG-1^+^) 7 days after rMVA immunization, while 20.5 ± 1.1% displayed this phenotype after rMVA-CD40L immunization. In the latter group, most cells were double positive for CD127 and KLRG-1 (55 ± 3%) – a population described to display an intermediate recall potential (50) and, recently, to form a highly protective effector-like memory subset (51). The percentage of memory precursor effector cells (MPECs, which are CD127^+^ KLRG-1^−^) and early effector cells (EECs, which are CD127^−^ KLRG-1^−^) was higher in rMVA immunized mice (43.8 ± 3.4 and 19.7 ± 2.3%, respectively). Thus, integration of CD40L into rMVA clearly influences cell proliferation and memory differentiation of CD8 T cells.

### Increased numbers and functionality of antigen-specific CD8 T cells after rMVA-CD40L immunization

**Figure 8:**
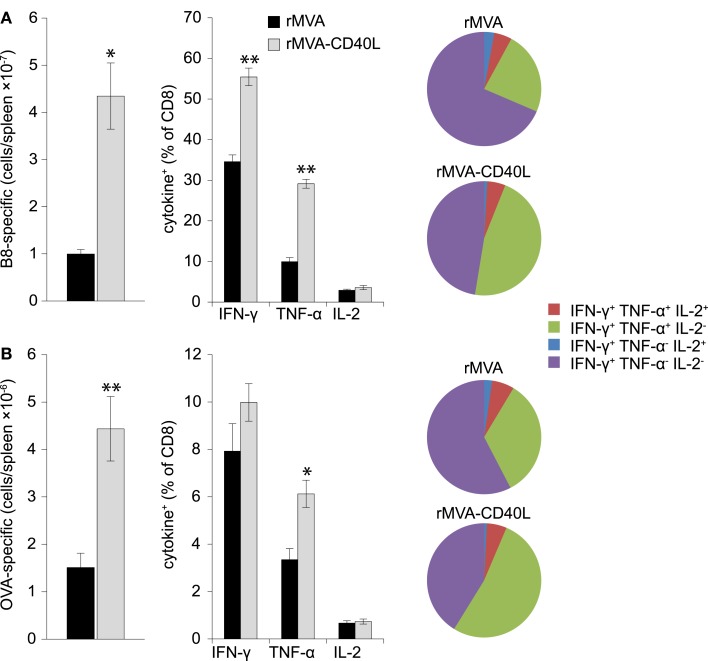
**Quantitative and qualitative improvement of primary CD8 T cell responses after immunization with rMVA-CD40L**. C57BL/6 mice were immunized with rMVA or rMVA-CD40L. B8- **(A)** and OVA- **(B)** specific CD8 T cell numbers in the spleen were determined by MHC I dextramer staining on day 7 after immunization (left panel). IFN-γ, TNF-α, and IL-2 production was analyzed by intracellular cytokine staining after standard 6 h *in vitro* re-stimulation with B8_20–27_ and OVA_257–264_ peptides (middle panel). Pie charts denote the fraction of IFN-γ^+^ cells expressing IFN-γ alone (violet), IFN-γ and TNF-α (green), IFN-γ and IL-2 (blue), and IFN-γ, TNF-α, and IL-2 (red). Note the increased percentage of IFN-γ/TNF-α double producer cells in rMVA-CD40L immunized mice (green area). Thus, in comparison to rMVA rMVA-CD40L immunization leads to functionally improved primary effector CD8 T cells. Data are representative of five mice per group and two independent experiments.

**Figure 9:**
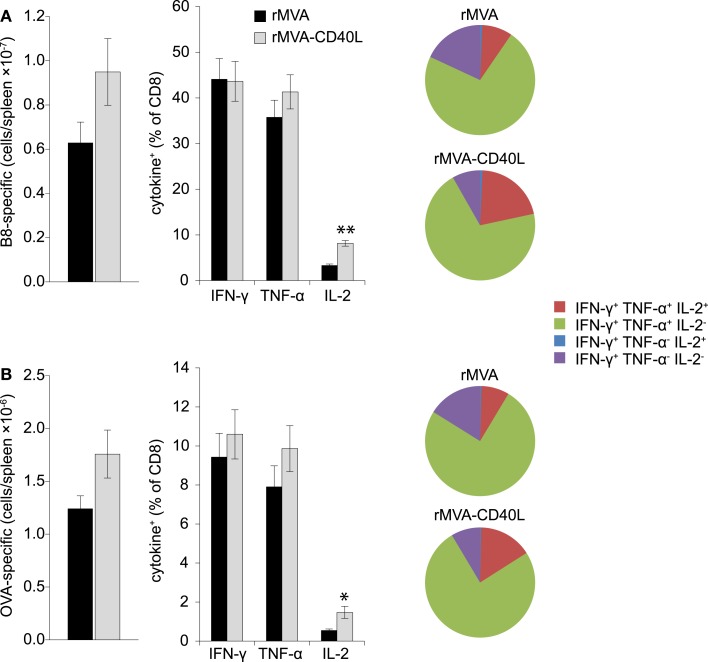
**Qualitative improvement of memory CD8 T cell responses after immunization with rMVA-CD40L**. C57BL/6 mice were immunized on day 0 and 35 with rMVA or rMVA-CD40L. B8- **(A)** and OVA- **(B)** specific CD8 T cell numbers in the spleen were determined by MHC I dextramer staining on day 104 (left panel). IFN-γ, TNF-α, and IL-2 production was analyzed by intracellular cytokine staining after standard 6 h *in vitro* re-stimulation with B8_20–27_ and OVA_257–264_ peptides (middle panel). Pie charts denote the fraction of IFN-γ^+^ cells expressing IFN-γ alone (violet), IFN-γ and TNF-α (green), IFN-γ and IL-2 (blue), and IFN-γ, TNF-α, and IL-2 (red). Note the increased percentage of IFN-γ/TNF-α/IL-2 triple producer cells in rMVA-CD40L immunized mice (red area). Thus, in comparison to rMVA rMVA-CD40L immunization leads to functionally improved memory CD8 T cells. Data are representative of five mice per group and two independent experiments.

T cell mediated protection is the result of increased numbers of antigen-specific CD8 T cells that are poised to rapidly secrete cytotoxic molecules (e.g., perforin, granzyme B) and cytokines (e.g., IFN-γ, TNF-α). Therefore, after having assessed increased frequencies after rMVA-CD40L immunization we set out to analyze the absolute number and functionality of effector and memory B8- and OVA-specific CD8 T cells in the spleen. To this end, B8- and OVA-specific CD8 T cells were enumerated by MHC class I dextramer staining and intracellular cytokine staining at day 7 after one immunization and at day 104 after two immunizations. Increased frequencies in the blood after rMVA-CD40L immunization translated into higher absolute numbers of B8- and OVA-specific CD8 T cells in the spleen at day 7 (Figures 8A,B, left panel). More IFN-γ and TNF-α producing effector CD8 T cells were also detected for both antigens (Figures 8A,B, middle panel). A higher functional capacity after rMVA-CD40L immunization was revealed by relative more IFN-γ^+^ TNF-α^+^ cells (Figures 8A,B, right panel). At this time point, there was no difference in IL-2 production between the two groups. Analysis of memory T cells at day 104 (=day 69 after the second immunization) revealed only marginal differences in absolute numbers of B8- and OVA-specific CD8 T cells between rMVA and rMVA-CD40L immunized mice (Figures 9A,B, left panel). Also, the frequencies of IFN-γ and TNF-α producing memory cells were similar (Figures 9A,B, middle panel). In contrast to effector cells, however, more rMVA-CD40L-induced memory CD8 T cells produced IL-2 upon re-stimulation with B8 and OVA peptides (Figures 9A,B, middle panel). Importantly, mice that were immunized with rMVA-CD40L had ∼2-fold more triple cytokine positive (IFN-γ, TNF-α, IL-2) antigen-specific CD8 T cells (Figures 9A,B, right panel). Those results demonstrated that immunization with rMVA-CD40L induced more multi-functional effector and memory CD8 T cells than immunization with rMVA. Since multi-functional T cells have been correlated with better protection against parasites, bacteria and viruses (9, 52–54) these data suggest that this vaccine vector might induce superior protection.

### Immunization with rMVA-CD40L confers protection independently of antibodies

**Figure 10:**
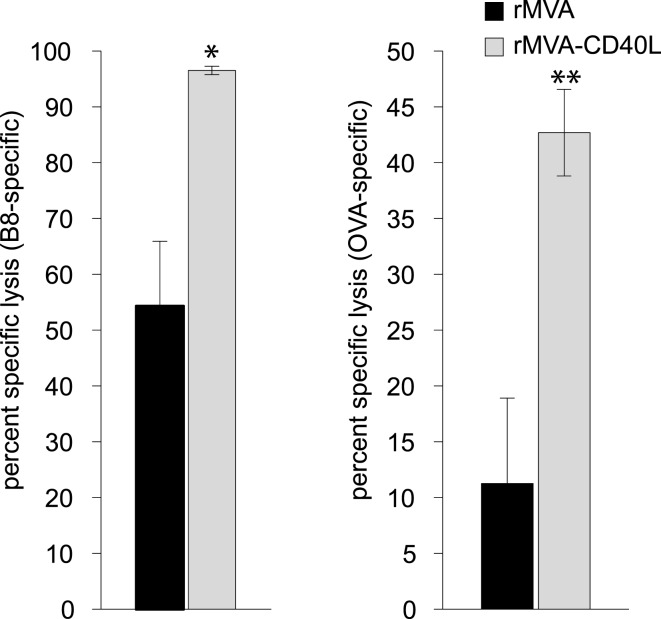
**Heightened CTL activity in rMVA-CD40L immunized mice**. In order to characterize the lytic activity of B8- and OVA-specific CD8 T cells, an *in vivo* CTL assay was performed on day 56 after immunization. Unpulsed (CFSElow), B8_20–27_-peptide pulsed (CFSEhigh) and OVA_257–264_-peptide pulsed (eFluor670high) splenic target cells were injected into rMVA and rMVA-CD40L immunized mice. Four hours post-transfer the ratio of unpulsed and peptide pulsed target cells was determined and the percent specific lysis calculated. Values represent mean ± SEM specific lysis of target cells of four mice per group. The experiment was performed twice with similar results.

In light of the astonishing strong CD8 T cell response after immunization with rMVA-CD40L we also wanted to evaluate whether immunization with this novel vaccine vector provides better protection in an infection model. First, we determined the cytolytic activity of antigen-specific memory CD8 T cells by an *in vivo* CTL assay at day 56 after a single immunization. The killing activity was analyzed 4 h after target cell transfer and revealed 54 compared to 97 percent specific lysis (PSL) of B8_20–27_-loaded target cells and 11 compared to 43 PSL of OVA_257–264_-loaded target cells for rMVA and rMVA-CD40L immunized mice, respectively (Figure 10).

**Figure 11:**
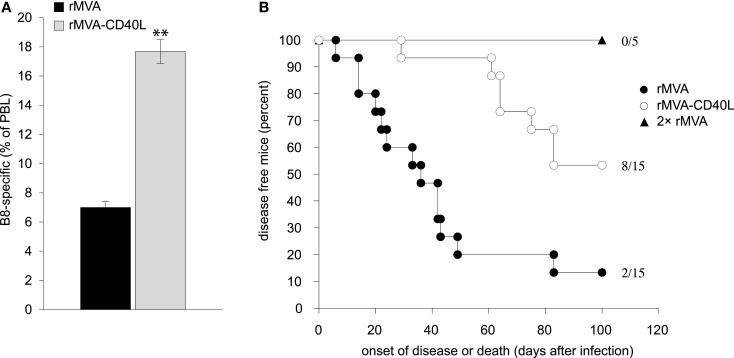
**Improved virus control in B cell deficient mice**. **(A)** In order to confirm that B cell deficient JHT mice used for challenge studies mount similar responses as C57BL/6 wt mice, JHT mice were immunized with rMVA and rMVA-CD40L. Seven days later the frequency of B8-specific CD8 T cells in the blood was determined by dextramer staining. Note that the percentage of B8-dextramer CD8 T cells after immunization is comparable between C57BL/6 wt mice and JHT mice (see Figure 6). Data are representative of five mice per group and two independent experiments. **(B)** JHT mice were immunized once with rMVA and rMVA-CD40L or twice with rMVA. Approximately 2 months after the first immunization, mice were infected with 1 × 10^5^ TCID_50_ ECTV and monitored daily for disease symptoms and survival. Shown is the day of disease onset or death. Disease onset was defined as the appearance of symptoms such as skin lesions, swollen limbs, and tail or, necrotic skin areas. The data are compiled from three independent experiments. The numbers depict the number of mice that remained symptom-free of the total number of mice/group.

In order to reveal the consequences of the improved CD8 T cell response after rMVA-CD40L immunization in a highly relevant infection model, we choose ectromelia virus (ECTV) infection of mice (mousepox). Mousepox is considered to be the best small animal model for human smallpox (38, 55). Because prophylactic immunization of wt mice with MVA already provides full protection against VV or ECTV challenge via antibodies (56), a T cell dependent and Ab independent challenge model was used (57, 58). To this end, B cell deficient JHT mice were immunized with rMVA or rMVA-CD40L and then challenged approximately 2 months later with a lethal dose of ECTV. B8-specific CD8 T cell responses were confirmed to be similar in JHT and wt mice (compare Figures 6 and 11A). While 87% of mice immunized with rMVA developed severe symptoms of ECTV infection, including necrotic tails, swollen and necrotic paws, and skin lesions, and either died or had to be euthanized, only 47% of the rMVA-CD40L immunized mice developed infection symptoms during an observation period of 100 days (Figure 11B). Also, the onset of disease was earlier in rMVA immunized than in rMVA-CD40L immunized mice. Because the frequency of peripheral B8-specific CD8 T cells was similar in mice that were immunized twice with rMVA and mice that were immunized only once with rMVA-CD40L (Figure 6), we tested whether JHT mice were protected after two rMVA immunizations. Indeed, those mice did not become symptomatic and remained healthy throughout the observation period. These data suggest that the heightened and qualitatively improved CD8 T cell response after a single rMVA-CD40L immunization leads to a better virus control after a lethal virus challenge even in the absence of antibodies.

## Discussion

It is generally agreed upon that protection against major human pathogens such as HIV, *Mycobacterium tuberculosis* and *Plasmodium falciparum* will require the induction of very large memory CD8 T cell responses. Despite some progress in recent years, so far no vaccines against these pathogens exist. It is this unmet medical need that let us design a recombinant vaccine vector targeted at enhancing both the magnitude and functionality of primary and memory CD8 T cell responses.

Pathogen-induced T cell responses start in secondary lymphoid organs such as lymphnodes and spleen, where naïve T cells are primed by antigen-presenting cells (APCs), preferentially DCs. Here, DCs present antigen via MHC class I and further provide co-stimulatory signals and inflammatory cytokines. The integration of these variegated signals is essential to achieve maximal T cell expansion and differentiation. Thus, the magnitude and functionality of responding CD8 T cells and the ensuing development of memory cells is largely programed in the first few days after pathogen encounter by tightly controlled DC-T cell interactions (17). After priming, CD8 T cells continue to encounter a plethora of signals that further shape their fate (18). Many of the factors that influence CTL responses have been elucidated in recent years, providing vaccinologists with more tools and knowledge for a rational development of therapeutics and vaccines.

In this study, we used the TNFSF member CD40L for genetic adjuvantation of a recombinant MVA vaccine vector. The strong stimulatory activity of CD40/CD40L in innate and adaptive immune responses makes CD40L a prime candidate for this purpose. Recombinant CD40L-encoding vaccinia viruses were already generated in the mid-1990s by Ruby et al. (59). This study, however, was designed to study the immunobiology of CD40L and not its applicability as a vaccine component. Based on the finding that nude and SCID mice could clear an infection with CD40L-expressing VV largely independently of IFN-γ and TNF-α, Ruby et al. postulated an antiviral activity for CD40L itself. While this first study focused on virus clearance, more recent studies investigated the immune properties of CD40L-adjuvanted poxvirus vectors *in vitro* and *in vivo* (34, 60, 61). The results from Liu et al. showed that co-immunization of ALVAC-HIV-1 (an attenuated canarypox vector) and a CD40L-expressing ALVAC led to 2-fold increased HIV-1-specific CD8 T cell responses in mice (61). Similar to this study, we could enhance vector- (B8_20–27_) and transgene- (OVA_257–264_ and Zaire Ebola virus GP_577–584_) specific CD8 T cell responses by co-immunization of rMVA and anti-CD40 mAb. In contrast to the study by Liu et al., we observed strong CD8 T cell responses already after a single immunization, even with non-adjuvanted rMVA. As shown before, OVA protein and anti-CD40 co-immunization did not lead to a detectable primary CTL response (22).

Because we hypothesized that the adjuvant effect is strongest when CD40L and the transgene are expressed synchronously by the same cell, we went on to construct a recombinant MVA with OVA and CD40L under the control of early/late promoters. It was shown previously that early transgene expression is necessary for the induction of optimal CTL responses (62). Initial *in vitro* analyses showed that integration of CD40L into rMVA drastically enhanced expression of CD80 and CD86 by FLDCs. Additionally, FLDCs transduced with rMVA-CD40L produced more IL-6 and IFN-α and secreted TNF-α and IL-12p70, cytokines that were not detected in the supernatant of rMVA treated FLDCs. These data are in line with previous studies with UV/psoralen-inactivated VV (34, 60) and ALVAC (61). In contrast to Bereta et al. (34), we did not find evidence for CD40L protein in viral particles (Figure 5). The reason for this discrepancy is not clear, but could be due to differential virus purification protocols that might influence the final composition of the virus suspension (63), or differences between VV and MVA.

Importantly, our *in vitro* findings could be directly translated *in vivo*, with increased DC activation and heightened cytokine secretion after i.v. immunization. Especially with the strong expression of CD80/86 (signal 2) by splenic DCs and the high amount of IL-12p70 (signal 3) rMVA-CD40L immunization induced an optimal milieu for CD8 T cell priming and memory formation. Indeed, quality and quantity of rMVA-CD40L induced CD8 T cell responses were superior to those induced by rMVA immunization. Therefore, it seems likely that the cooperative effect of CD80/86 and IL-12 co-stimulation is largely responsible for the enhanced CTL response. The synergistic effect of both molecules was demonstrated *in vitro* (64) and also in an experimental antitumor model in mice using recombinant VV (65, 66). A further potent co-stimulatory molecule, namely CD70, was also up-regulated on splenic DCs after rMVA-CD40L immunization (data not shown). Because rMVA-CD40L immunization leads to both, high levels of systemic IL-12 and strong up-regulation of CD80/86, it obviates the need for exogenous IL-12 administration or the construction of CD80, IL-12p35, IL-12p40 triple recombinants as done previously in the studies by Carroll et al. and Rao et al. (65, 66). Another possible explanation for the enhanced immunogenicity of rMVA-CD40L could be a direct interaction between CD40 on CD8 T cells and CD40L expressed by DCs, as described by Johnson et al. (32).

In recent years, a highly inflammatory milieu was correlated with enhanced formation of short lived effector cells (SLECs, CD127^−^ KLRG-1^+^) at the expense of memory precursor effector cells (MPECs, CD127^+^ KLRG-1^−^), thus limiting the amount of long lived memory cells (49, 50, 67). Detailed studies revealed this differentiation to be dependent on the ratio between the transcription factors T-bet and Eomesodermin (Eomes) (49, 68). CD80 co-stimulation and IL-12 signaling were revealed to be major drivers of enhanced T-bet expression via the mTOR pathway (69). Since we detected higher expression of CD80, CD86, and IL-12p70 by DCs stimulated by rMVA-CD40L *in vitro* and *in vivo*, we expected a shift of primary CTLs toward a SLEC phenotype. Surprisingly, antigen-specific CD8 T cells at day 7 after rMVA-CD40L immunization were largely CD127^+^ KLRG-1^+^, whereas the largest CTL population after rMVA immunization was CD127^+^ KLRG-1^−^ (Figure 7C). Thus, the priming under strong stimulatory and inflammatory conditions (i.e., after rMVA-CD40L immunization) led to more KLRG-1^+^ CD8 T cells but did not repress CD127 expression, because B8- and OVA-specific CD8 T cells had higher surface expression of CD127 after rMVA-CD40L than after rMVA immunization (Figures 6 and 7). Interestingly, CD127^+^ KLRG-1^+^ CD8 T cells are usually found in secondary and tertiary memory responses after multiple infections or immunizations (50, 70). KLRG-1^+^ CD8 T cells were initially described to have a severely diminished potential to proliferate after antigen re-encounter (71). Adding CD127 for further subdivision of KLRG-1^+^ cells, Obar et al. described an intermediate recall potential for CD127^+^ KLRG-1^+^ double positive CD8 T cells, whereas CD127^−^ KLRG-1^+^ cells were most drastically impaired in their ability to expand after Ag stimulation (50). Interestingly, Olson et al. described recently an effector-like memory subset with a CD27lo CD43lo CD62Llo KLRG-1hi CD127int phenotype (51). This CD8 T cell subset displayed enhanced capacity to eliminate *Listeria* and was preferentially localized to the red pulp of the spleen. It will be interesting to test the CD127^+^ KLRG-1^+^ CD8 T cells induced by rMVA-CD40L immunization for the expression of CD27 and CD43 in order to find out whether they resemble the cells described by Olson et al. Besides inflammation (i.e., IL-12) the expression of CD25 and sustained IL-2 signaling during expansion were linked to the development of SLECs (48, 72, 73). Therefore, our data showing higher CD25 expression on B8-specific CD8 T cells after rMVA-CD40L immunization would, once again, favor the development of SLECs. The dominance of CD127^+^ KLRG-1^+^ double positive CD8 T cells at the peak of the primary response after rMVA-CD40L immunization is probably a result of the combination of the high levels of IL-12p70 and enhanced IL-2 signaling. In the defined cell-culture setting used by Pipkin et al. inflammatory signals (i.e., CpG and IL-12) enhanced expression of CD25 and T-bet but counteracted IL-2-mediated repression of CD127 (73). The higher CD25 expression on B8-specific CD8 T cells in rMVA-CD40L-immunized mice, in comparison to rMVA immunized mice, is a likely explanation for more and faster proliferating T cells in this group, because IL-2 signaling is directly linked to sustained proliferative expansion of CD8 T cells (46–48). Taken together, our data support a model in which combined TLR- and CD40-stimulation of DCs after encounter with rMVA-CD40L increases the expression of CD80/CD86 co-stimulatory molecules and IL-12p70. Signaling via the CD28:CD80/86 pathway then causes IL-2 expression perhaps by CD4 T cells (74), which regulates CD25 expression on CD8 T cells (75). The early expression of IL-12p70 in our experiments and its demonstrated linkage with TCR engagement (76) suggest an influence of this cytokine on effector T cell generation during the initial priming phase. IL-2, in contrast, exerts its effect on T cell differentiation at later times. The integration of all signals finally leads to higher numbers of antigen-specific CD8 T cells with enhanced functionality and a CD127^+^ KLRG-1^+^ phenotype after rMVA-CD40L immunization.

Remarkably, both, the number and phenotype of B8-specific CD8 T cells are similar after two immunizations with rMVA and a single immunization with rMVA-CD40L (Figures 6 and 7 and data not shown). We cannot say whether primary rMVA-CD40L-induced effector T cells really resemble secondary rMVA-induced effector T cells in more than the investigated parameters. The challenge studies with ECTV at least suggest that, in contrast to a single rMVA immunization, a high degree of CTL-mediated protection can be achieved by a single rMVA-CD40L-immunization, indicative of a more efficacious CTL response (Figure 11). Whether this is the result of higher numbers or enhanced functionality or both remains to be determined. Furthermore, it would be very interesting to perform detailed “omical” analyses of the CD8 T cell populations in rMVA- and rMVA-CD40L-immunized mice in order to reveal the entire impact rMVA-CD40L-immunization has on the CD8 T cell response.

The fast generation of high numbers of polyfunctional CTLs with characteristics of secondary effector cells by rMVA-CD40L immunization offers clear advantages over non-adjuvanted vectors: (1) lower vaccine doses can be used or fewer vaccinations performed to reach a similar level of protection; (2) protective immunity can be established faster – a feature that is important in emergency settings such as bioterrorism attacks or sudden outbreaks of disease; (3) protective levels of CTLs might be achievable with such a vaccine vector even in situations where very high numbers of polyfunctional CTLs are likely to be required [e.g., *P. falciparum* (14, 15) and HIV/SIV (10, 77)]; (4) the combination of strong innate and adaptive immune responses might allow therapeutic vaccinations against tumors or chronic infections, where high hurdles such as tolerance or immune inhibitory conditions must be overcome (78).

Overall, we believe that recombinant MVA vaccines genetically adjuvanted with CD40L are a major step forward the development of vaccines against difficult targets that require particular high and efficient CTL responses for protection.

## Materials and Methods

### Ethics statement

All animal experiments were approved by the animal ethics committee of the government of Upper Bavaria (Regierung von Oberbayern, Sachgebiet 54, Tierschutz) and were carried out in accordance with the approved guidelines for animal experiments at Bavarian Nordic GmbH.

### Mice

Mice were bred and maintained either in the animal facilities at Bavarian Nordic GmbH or at the University of Zurich according to institutional guidelines. C57BL/6J (H-2b) and CBA/J (H-2k) mice were purchased from JANVIER LABS. MHC class II deficient (MHC II^−/−^), CD40 deficient (CD40^−/−^), CD40L deficient (CD40L^−/−^), and JHT mice were on a C57BL/6 background and were obtained from the animal facility of the University Zurich.

### Generation of MVA-BN recombinants

All recombinant virus vectors used for this study were based on a cloned version of MVA-BN^®^ in a bacterial artificial chromosome (BAC). MVA-BN^®^ was developed by Bavarian Nordic and is deposited at the European Collection of Cell Cultures (ECACC) (V00083008). The generation of MVA recombinants was carried out as described recently (40). Briefly, the sequence of the strong synthetic early late pS promoter comprises 40 nucleotides exactly matching the previously described sequence (41). The pS promoter was cloned upstream of the open reading frame for chicken OVA. The pHyb promoter was developed and described by Baur et al. (40) and comprises a late element from the promoter directing the expression of the ATI protein in cowpox virus (79, 80) and five tandemly arranged early elements derived from a modified p7.5 promoter (81). The pHyb promoter was cloned upstream of the open reading for murine CD40L. Infectious viruses were reconstituted from BACs by transfecting BAC DNA into BHK-21 cells and superinfecting them with Shope fibroma virus as a helper virus. After three additional passages on primary chicken embryo fibroblasts (CEF), helper virus free MVA-OVA, MVA-OVA-CD40L, and MVA-ZEBOV-GP viruses were obtained. All viruses used in animal experiments were purified twice through a sucrose cushion. For the UV-inactivation of viruses concentrated virus stocks were UV irradiated with an UV Chamber (Genelinker GS, Bio-Rad laboratories, Munich Germany) in the presence of psoralen.

### Immunization of mice

Intravenous injections were given into a lateral tail vein with a total volume of 200 μl containing 5 × 10^7^ TCID_50_ of the respective MVA recombinants. Where noted, 50 μg anti-CD40 Ab (Bio X Cell) were mixed with MVA recombinants or 100 μg EndoGrade Ovalbumin (Hyglos) prior to injection. For ECTV (strain Moscow) infection, mice were anesthetized with ketamine/xylazine and virus (1 × 10^5^ TCID_50_) was applied by intranasal (i.n.) drop wise installation in a total volume of 50 μl. The health status of infected mice was checked daily.

### Flow cytometry

Mononuclear cell suspensions were stained with appropriate dilutions of the following monoclonal antibodies: anti CD3-FITC, CD3-PECy7, CD4-APC-H7, CD8α-FITC, CD8α-PerCP-Cy5.5, CD19-FITC, CD19-PerCP-Cy5.5, CD44-FITC, CD62L-FITC, CD69-FITC, CD80-FITC, CD80-PE, CD86-FITC, NK1.1-FITC, NK1.1-APC, NK1.1-PerCP-Cy5.5, IL-2-APC, TNF-α-PE (all BD Biosciences), CD4-Alexa700, CD3-PerCP-Cy5.5, CD8α-Alexa700, CD8α-eFluor450, CD11b-Alexa700, CD11c-PECy7, CD25-PE, CD44-PerCP-Cy5.5, CD44-APC-eFluor780, CD45R (B220)-eFluor780, CD86-eFluor605, CD127-PECy7, CD154 (CD40L)-eFluor780, H-2Kb/SIINFEKL-APC (25-D1.16), KLRG-1-PerCP-eFluor710, KLRG-1-FITC, IFNγ-PECy7, Bcl-2-FITC (all eBioscience), and anti-Granzyme B-PE (GRB04, Life Technologies). APC-conjugated MHC class I H-2Kb dextramers loaded with B8_20–27_-peptide (TSYKFESV) or PE-conjugated MHC class I H-2Kb dextramers loaded with OVA_257–264_-peptide (SIINFEKL) were used according to the manufacturer’s instructions (Immudex). For intracellular cytokine staining, cells were incubated with 2.5 μg/ml of MHC class I restricted peptides [B8_20–27_, OVA_257–264_, or ZEBOV-GP_577–584_ (TELRTFSI)] for 5–6 h at 37°C in complete RPMI in the presence of 1 μl/ml GolgiPlug (BD Biosciences). Peptides were purchased from GenScript. Intracellular staining of IFN-γ, TNF-α, and IL-2 was performed after fixation/permeabilization according to the manufacturer’s instructions (BD Cytofix/Cytoperm, BD Biosciences). In order to measure cell proliferation 100 μg EdU were injected i.v. into MVA immunized animals. Spleens were removed 1 h later for surface staining and subsequent EdU staining according to the manufacturer’s instructions (Click-iT EdU Alexa Fluor488 Flow cytometry Assay Kit, Life Technologies). For live/dead discrimination cells were stained before fixation according to the manufacturer’s instructions (LIVE/DEAD fixable violet dead cell staining kit, Life Technologies). All cells were acquired using a digital flow cytometer (LSR II, BD Biosciences) and data were analyzed with FlowJo software (Tree Star).

### *In vivo* CTL assay

The *in vivo* CTL assay was essentially performed as described before (82) but extended to two target cell populations. Briefly, mononuclear spleen cell suspensions from naïve mice were incubated at ∼2 × 10^7^ cells/ml with 20 μg/ml B8_20–27_-peptide, OVA_257–264_-peptide, or without peptide for 1 h at 37°C. Each spleen cell population was labeled with 5 μM CFSE (Life Technologies), 0.5 μM CFSE or 5 μM eFluor670 (eBioscience) for 10 min at 37°C. Labeling was stopped by addition of 10 ml FCS. Washed cells were mixed at a 1:1:1 ratio and 1–2 × 10^7^ cells were injected i.v. into syngeneic naïve and immunized mice. Mice were sacrificed after 4 h and mononuclear spleen cell suspensions prepared. Cells were analyzed flow cytometrically. PSL of fluorescent target cells was calculated as follows: ratio = percentage of unpulsed cells/percentage of pulsed cells; PSL = (1−ratio unprimed/ratio primed) × 100.

### *In vitro* DC stimulation

FLT3-L bone marrow culture derived DCs were prepared as described (83). For *in vitro* analysis of DC activation and gene expression, wt FLDCs were transduced with MVA-OVA or MVA-OVA-CD40L (MOI 2). Three, six, and nine hours later DCs were harvested and stained for flow cytometric analysis. Supernatant was kept for cytokine analysis.

### Cytokine detection

Cytokine concentrations in serum and supernatant of FLDC cultures were determined by FlowCytomix bead assay (eBioscience) according to the manufacturer’s instructions.

### Statistical analysis

Statistical significance was calculated using an unpaired, two-tailed Student’s *t*-test. *p*-Values of<0.05 were considered statistically significant (*) and *p*-values of<0.005 were considered highly statistically significant (**).

## Conflict of Interest Statement

All authors are employees of Bavarian Nordic GmbH, which is the founder of the study. The authors designed the study, collected and analyzed data, decided to publish and prepared the manuscript. The MVA used for this study was MVA-BN^®^. MVA-BN^®^ is Bavarian Nordic’s proprietary and patented technology.
